# *Pleopunctum xizangense* sp. nov. (Pleosporales, Phaeoseptaceae) and *Pseudolophiostoma yunnanense* sp. nov. (Pleosporales, Lophiostomataceae), and a new record of *Pleopunctum pseudoellipsoideum* on Fagales

**DOI:** 10.3897/mycokeys.131.175264

**Published:** 2026-04-16

**Authors:** Yan-Yan Yang, Tai-Shun Li, Vinodhini Thiyagaraja, Chitrabhanu S. Bhunjun, Shu-Cheng He, Pattana Kakumyan, Kevin D. Hyde, Qi Zhao

**Affiliations:** 1 State Key Laboratory of Phytochemistry and Natural Medicines, Yunnan Key Laboratory for Fungal Diversity and Green Development, Kunming Institute of Botany, Chinese Academy of Sciences, Kunming 650201, P.R. China. Center of Excellence in Fungal Research, Mae Fah Luang University Chiang Rai Thailand; 2 School of Science, Mae Fah Luang University, Chiang Rai 57100, Thailand Mae Fah Luang University Chiang Rai Thailand; 3 Center of Excellence in Fungal Research, Mae Fah Luang University, Chiang Rai 57100, Thailand Microbial Products and Innovations Research Group, Mae Fah Luang University Chiang Rai Thailand; 4 State Key Laboratory of Efficient Production of Forest Resources, School of Ecology and Nature Conservation, Beijing Forestry University, Beijing 100083, China Kunming Institute of Botany, Chinese Academy of Sciences Kunming China; 5 Microbial Products and Innovations Research Group, Mae Fah Luang University, Chiang Rai 57100, Thailand Beijing Forestry University Beijing China

**Keywords:** 2 new taxa, asexual morph, Dothideomycetes, phylogeny, taxonomy

## Abstract

Pleosporales is the largest order of Dothideomycetes and is widely distributed in terrestrial and aquatic environments. During a survey of saprobic fungi in southwestern China, we collected and isolated some Pleosporales taxa. Based on morphological comparisons and multi-gene (SSU-ITS-LSU-*tef1*) phylogenetic analyses, we describe two new species, *Pleopunctum xizangense* and *Pseudolophiostoma yunnanense*, as well as a new host record, *Pleopunctum pseudoellipsoideum*, that were collected from the plant family Fagales. *Pseudolophiostoma yunnanense* was found in its asexual state, the first such account for this genus. In this study, we compare and discuss morphologically similar species and phylogenetically related taxa, providing comprehensive illustrations and descriptions of the isolates.

## Introduction

Luttrell (1955) invalidly established Pleosporales to accommodate seven families. Subsequently, Barr (1987) formally established Pleosporales in Dothideomycetes with *Pleospora* Rabenh. ex Ces. & De Not. (= *Stemphylium* Wallr) as the type genus, and recognised 18 families. In recent years, more families have been accepted in this order (Wijayawardene et al. 2020; Hongsanan et al. 2020). Pleosporales is the largest order of Dothideomycetes, and it contains 94 families, with more than 400 genera (Yang et al. 2022; Hyde et al. 2024). Pleosporales species are widely distributed in terrestrial and aquatic habitats worldwide (Yang et al. 2022; Xu et al. 2023), and can be hyperparasites on fungi or insects, epiphytes or endophytes of living leaves or woods, lichenised or saprobes of dead plant leaves, woods or bark (Ramesh 2003; Kruys et al. 2006; Hyde et al. 2020). These species have been recorded on a variety of hosts, such as *Coffea arabica* (coffee), *Mangifera indica* (mango), *Zanthoxylum schinifolium*, *Mangifera* sp., *Brassica* sp., and *Clematis vitalba* (Lu et al. 2022; Xu et al. 2022; Phukhamsakda et al. 2022; Yang et al. 2022; Hu et al. 2023; Madagammana et al. 2024). The members of the order are characterised by sexual and asexual morphs. The sexual morph is characterised by ostiolar, pseudothecial ascomata, occasionally with a papillate apex, bitunicate and fissitunicate asci with an ocular chamber or apical ring, septate ascospores with or without a gelatinous sheath, while the asexual morph is coelomycetous or hyphomycetous (Zhang et al. 2009; Su et al. 2022).

Based on phylogenetic analysis, Hyde et al. (2018) introduced Phaeoseptaceae to accommodate *Lignosphaeria*, *Neolophiostoma*, and *Phaeoseptum* (type genus). The sexual morph of Phaeoseptaceae is characterised by immersed, short papillate ascomata with an apical ostiole, cylindrical-clavate, bitunicate, long pedicellate asci with a small ocular chamber, and muriform, multi-septate, light brown ascospores (Hyde et al. 2018; Liu et al. 2019). The asexual state is hyphomycetous, with punctiform colonies, macronematous and branched conidiophores with septate, monoblastic conidiogenous cells, and muriform, acrogenous, broadly oval to ellipsoidal conidia often with a hyaline basal cell (Liu et al. 2019). *Pleopunctum* was introduced by Liu et al. (2019) as the first hyphomycetous genus in Phaeoseptaceae.

Lophiostomataceae was first reported by Nitschke (1869) and formally established by Saccardo (1883), with *Lophiostoma macrostomum* (Tode) Ces. & De Not. as the type genus. Presently, Lophiostomataceae encompasses 30 genera, and is characterised by immersed to erumpent ascomata, slit-like ostioles with a small to large, fissitunicate, bitunicate asci with furcate pedicel, and septate, hyaline to dark brown ascospores with mucilaginous sheaths or terminal appendages (Tibpromma et al. 2017; Brahmanage et al. 2020). Species of Lophiostomataceae occur on herbs and woody plants in terrestrial and aquatic environments as saprobic (Holm and Holm 1988; Mugambi and Huhndorf 2009). Lophiostomataceae and Lophiotremataceae are highly similar in morphology. Andreasen et al. (2021) resolved the phylogenetic relationships within these two families by obtaining additional molecular evidence.

*Pleopunctum* was established by Liu et al. (2019) to accommodate two species, namely, *P. ellipticum* (type species) and *P. pseudoellipticum*. *Pleopunctum* is the first hyphomycetous genus in Phaeoseptaceae (Liu et al. 2019). The genus *Pleopunctum* includes 11 species, all reported only in asexual morphs, and is found in Thailand and China, while some species have been reported from freshwater habitats (Xu et al. 2023; Zhang et al. 2023). *Pleopunctum* is characterised by gregarious, sporodochial colonies, macronematous, mononematous, septate conidiophores, monoblastic and terminal conidiogenous cells, multi-septate, brown, muriform and oval to ellipsoidal conidia often with a hyaline, elliptical to globose basal cell (Liu et al. 2019; Xu et al. 2023). In addition, some species of *Pleopunctum* have been reported with hyaline phragmosporous or dictyosporous conidia (Wanasinghe et al. 2022).

Thambugala et al. (2015) established the new genus *Pseudolophiostoma* in Lophiostomataceae, with *Pseudolophiostoma vitigenum* as the type species, based on phylogenetic evidence. This species was originally described as *Lophiotrema vitigenum* by Tanaka and Harada (2003) and was subsequently transferred to *Lophiostoma* by Hirayama and Tanaka (2011) based on phylogenetic analysis and morphological characteristics. *Pseudolophiostoma* species have been collected from China, Japan, and Thailand. They are typically saprobic on a wide range of herbaceous, woody, or climbing plants, and reported host substrates include *Bidens pilosa* var. *radiata*, *Castanopsis calathiformis*, *Clematis fulvicoma*, *Livistona boninensis*, *Stachytarpheta jamaicensis*, and *Vitis coignetiae* (Thambugala et al. 2015; Hashimoto et al. 2018; Tennakoon et al 2018; Ren et al. 2024). Only the sexual morph of *Pseudolophiostoma* species has been reported so far, and its characteristics are immersed ascomata with an elongated ostiolar neck, septate, branched pseudoparaphyses, bitunicate, fissitunicate asci, fusiform, 1-septate ascospores with a narrow bipolar sheath.

During a recent investigation of microfungal diversity in Yunnan Province and Xizang Autonomous Region, China (Xu et al. 2024, 2025; Lu et al. 2025), three saprobic fungi were encountered on dead twigs of Fagales trees. In China, Fagales species are widely distributed in the northern and southern regions, with four families, namely, Betulaceae, Fagaceae, Juglandaceae, and Myricaceae, particularly abundant in Yunnan Province (Feng and Yang 2018; Yang et al. 2023). Various microfungi have been identified on the leaves, branches, and roots of Fagaceae (Jayawardena et al. 2022; Jiang et al. 2022; Phukhamsakda et al. 2022). In the present study, based on morphological and phylogenetic analyses, we introduce two new species and a new host record within Pleosporales.

## Materials and methods

### Sample collection, morphological study, and isolation

Fresh materials were collected from dead wood at different locations in southwestern China in 2022. Information for each sample was recorded, including host, collection site, collection date, and collector. All samples were placed in plastic bags and brought to the laboratory. Materials were examined using a stereomicroscope (Model C-PSN Nikon Corporation, Tokyo, Japan). Fruiting bodies were picked with a needle and mounted in distilled water for microscopic analysis. The micromorphological structures of conidiophores, conidiogenous cells, and conidia were observed using a Nikon ECLIPSE Ni compound microscope (Model Eclipse Ni-UNikon Corporation, Tokyo, Japan), and photomicrographs were obtained using a charge-coupled device camera (IMG SC2000C, Jiangsu Province, China). Photo plates were edited with Adobe Photoshop 2019, and measurements were taken with the Tarosoft (R) Image Frame Work program. The method by Senanayake et al. (2020) was used for single-spore isolation to obtain a pure culture. Germinated spores were observed under a stereo microscope (Model Mshot ML31, China), transferred to a fresh potato dextrose agar (PDA; 39 g/L distilled water, Difco potato dextrose) plate, and then incubated at 20 °C for one month. All living cultures were deposited in the Culture Collection of the Herbarium of Cryptogams, Kunming Institute of Botany, Academia Sinica (KUNCC), and the herbarium materials were deposited in Kunming Institute of Botany, Academia Sinica (HKAS). The Faces of Fungi (FoF) number was obtained following Jayasiri et al. (2015), and the Index Fungorum number was registered in Index Fungorum (http://www.indexfungorum.org).

### DNA extraction, amplification, and sequencing

Fresh mycelia were extracted from the culture for DNA sequencing. Genomic DNA was extracted using a TreliefTM Plant Genomic DNA extraction kit (TsingKe, Beijing, China) according to the manufacturer’s protocols. The partial nuclear genes were subjected to PCR amplification and sequencing: large subunit (LSU), internal transcribed spacer (ITS), 18S ribosomal RNA (SSU), and translation elongation factor 1-alpha (*tef1*). The primers of each locus were LR0R and LR5 for LSU (Vilgalys and Hester 1990), ITS4 and ITS5 for ITS (White et al. 1990), NS1 and NS4 for SSU (White et al. 1990), and 983F and 2218R for *tef1* (Rehner and Buckley 2005). The total final volume of PCR mixtures was 25 μl, which contained 1 μl of each forward and reverse primer, 2 μl of DNA template and 21 μl 2 × PCR Master Mix. The following thermo-cycling parameters were used for the ITS, LSU, SSU, and *tef1* regions: initially, 95 °C for 3 min, followed by 35 cycles of denaturation at 95 °C for 15 s, annealing at 54 °C for 15 s, elongation at 72 °C for 20 s, and final extension at 72 °C for 5 min. Purification and sequencing of PCR products were performed at Tsingke (Kunming, China), and the sequences were submitted to GenBank for accession numbers.

### Sequence alignment and phylogenetic analyses

The newly generated sequences were assembled with Sequencing Project Management (SeqMan) (Clewley 1995). BLAST searches are performed on the resulting sequences to check for contamination and to identify relevant taxa for analysis (http://blast.ncbi.nlm.nih.gov/). Sequence data were downloaded from GenBank in accordance with recent publications (Table 1). Each gene locus was automatically aligned with MAFFT (http://mafft.cbrc.jp/alignment/server/, Katoh et al. 2019) and trimmed with trimAl v1.2rev59 (Castresana 2000; Talavera and Castresana 2007). Single-gene alignments were combined using Sequence Matrix 1.7.8 (Vaidya et al. 2011).

**Table 1.** Taxa names, strain numbers, and corresponding GenBank accession numbers of the taxa used in the phylogenetic analyses in this study.

Taxa	Strain number	GenBank Accession Numbers			
		LSU	ITS	SSU	*tef1*
*Alpestrisphaeria jonesii*	GAAZ 54-1	KX687753	KX687757	KX687755	KX687759
*Alpestrisphaeria jonesii*	GAAZ 54-2	KX687754	KX687758	KX687756	KX687760
*Alpestrisphaeria terricola*	SC-12^T^	JX985750	JN662930	JX985749	–
*Biappendiculispora japonica*	KT 573^T^	AB619005	LC001728	AB618686	LC001744
*Biappendiculispora japonica*	KT 794	AB619007	LC001730	AB618688	LC001746
*Biappendiculispora japonica*	KT 686	AB619006	LC001729	AB618687	LC001745
*Capulatispora sagittiforme*	KT 1934^T^	AB369267	AB369268	AB618693	LC001756
*Coelodictyosporium muriforme*	MFLUCC 13-0351^T^	KP888641	KP899136	KP899127	KR075163
*Coelodictyosporium pseudodictyosporium*	MFLUCC 13-0451^T^	KR025862	KR025858	–	–
*Crassiclypeus aquaticus*	KT 970^T^	LC312530	LC312501	LC312472	LC312559
*Crassiclypeus aquaticus*	KH 104	LC312528	LC312499	LC312470	LC312557
*Decaisnella formosa*	BCC 25617	GQ925847	–	–	GU479850
*Decaisnella formosa*	BCC 25616	GQ925846	–	–	GU479851
*Desertiserpentica hydei*	SQUCC 15092^T^	MW077156	MW077147	MW077163	MW075773
*Dimorphiopsis brachystegiae*	CPC 22679^T^	KF777213	KF777160	–	–
*Flabellascoma aquaticum*	KUMCC 15-0258	MN274564	MN304827	MN304832	MN328898
*Flabellascoma cycadicola*	KT 2034^T^	LC312531	LC312502	LC312473	LC312560
*Flabellascoma fusiforme*	MFLUCC 18-1584	MN274567	MN304830	–	MN328902
*Guttulispora crataegi*	MFLUCC 14-0993	KP888640	KP899135	KP899126	KR075162
*Guttulispora crataegi*	MFLUCC 13-0442^T^	KP888639	KP899134	KP899125	KR075161
*Kiskunsagia ubrizsyi*	REF121^T^	MK589359	JN859341	MK589351	MK599325
*Lentistoma aquaticum*	MFLUCC 18-1275	MN913723	MT627697	MT864320	MT954370
*Lentistoma bipolare*	KT 3056	LC312542	LC312513	LC312484	LC312571
*Lentistoma bipolare*	CBS 115375	LC312535	LC312506	LC312477	LC312564
*Leptoparies magnoliae*	MFLU 18-1291	ON870390	ON878077	ON870915	–
*Leptoparies palmarum*	KT 1653^T^	LC312543	LC312514	LC312485	LC312572
*Leptosphaeria heterospora*	AFTOL-ID 1036	AY016369	GQ203795	–	DQ497609
*Lignosphaeria diospyrosa*	MFLU 17-1543	MT221674	–	–	MT221676
*Lignosphaeria fusispora*	MFLUCC 11-0377	NG_069269	NR_164233	–	–
*Lignosphaeria thailandica*	MFLUCC 11-0376	NG_069268	NR_164232	–	–
*Lophiohelichrysum helichrysi*	IT-1296^T^	KT333436	KT333435	KT333437	KT427535
*Lophiomurispora hongheensis*	KUMCC 20-0217	NG_075404	NR_172442	NG_074965	MW256817
*Lophiopoacea paramacrostomum*	MFLUCC 11-0463^T^	KP888636	–	KP899122	–
*Lophiopoacea winteri*	KT 740	AB619017	JN942969	AB618699	LC001763
*Lophiopoacea winteri*	KT 764	AB619018	JN942968	AB618700	LC001764
*Lophiostoma semiliberum*	KT 622	AB619012	JN942966	AB618694	LC001757
*Lophiostoma semiliberum*	KT 652	AB619013	JN942967	AB618695	LC001758
*Neopaucispora rosae-ecae*	MFLUCC 17-0807^T^	MG829033	MG828924	MG829139	MG829217
*Neotrematosphaeria biappendiculatum*	KTC 975	GU205228	–	GU205254	–
*Neotrematosphaeria biappendiculatum*	KTC 1124^T^	GU205227	–	GU205256	–
*Neovaginatispora clematidis*	MFLUCC 17-2149	MT214559	MT310606	MT226676	MT394738
*Neovaginatispora fuckelii*	MFLUCC 17-1334	MN274565	MN304828	MN304833	MN328899
*Neovaginatispora fuckelii*	KT 634	AB619009	LC001732	AB618690	LC001750
*Oleaginea sichuanensis*	CGMCC 3.24427	OR253236	OR253084	–	OR262145
*Oleaginea sichuanensis*	UESTCC 23.0052	OR253237	OR253085	–	OR262147
*Paramonodictys dispersa*	KUNCC 10788	OQ146988	ON261165	–	OQ943185
*Paramonodictys yunnanensis*	KUNCC 21-0037	OL436226	OL436231	–	OL505585
*Parapaucispora pseudoarmatispora*	KT 2237	LC100026	LC100021	LC100018	LC100030
*Paucispora kunmingense*	MFLUCC 17-0932^T^	MF173428	MF173432	MF173430	MF173434
*Paucispora quadrispora*	KT 843T	AB619011	LC001734	AB618692	LC001755
*Phaeoseptum aquaticum*	CBS 123113	JN644072	–	–	–
*Phaeoseptum carolshearerianum*	NFCCI-4221^T^	MK307813	MK307810	–	MK309874
*Phaeoseptum carolshearerianum*	NFCCI-4384	MK307815	MK307812	–	MK309876
*Phaeoseptum hydei*	MFLUCC 17-0801^T^	MT240623	MT240622	–	MT241506
*Phaeoseptum mali*	MFLUCC 17-2108	MK625197	MK659580	–	MK647990
*Phaeoseptum mali*	HKAS122917	ON009099	ON009115	–	ON009258
*Phaeoseptum manglicola*	NFCCI-4666^T^	MK307814	MK307811	–	MK309875
*Phaeoseptum terricola*	MFLUCC 10–0102	MH105779	–	–	MH105781
*Platystomum crataegi*	MFLUCC 14-0925^T^	KT026109	KT026117	KT026113	KT026121
*Platystomum rosae*	MFLUCC 15-0633^T^	KT026111	KT026119	KT026115	–
*Platystomum salicicola*	MFLUCC 15-0632^T^	KT026110	KT026118	KT026114	–
*Pleopunctum guizhouense*	GZCC 23–0595	OR091332	OR098710	–	–
*Pleopunctum megalosporum*	KUNCC 10785	OQ146985	ON261162	–	OQ943186
*Pleopunctum megalosporum*	KUNCC 10442	OQ146986	OQ135180	–	OQ943187
*Pleopunctum pseudoellipsoideum*	MFLU 19–0686	MK804518	MK804513	–	MK828511
*Pleopunctum pseudoellipsoideum*	KUMCC 21–0820	ON009100	ON009116	–	ON009259
*Pleopunctum pseudoellipsoideum*	HKAS122915	ON009101	ON009117	–	ON009260
*Pleopunctum rotundatum*	KUNCC 10787	OQ146987	ON261164	–	OQ943194
*Pleopunctum rotundatum*	KUNCC 10780	OQ146980	ON261157	–	OQ943193
*Pleopunctum baoshanense*	KUNCC 21-0494^T^	PP779902	PP779893	–	PP778367
*Pleopunctum bauhiniae*	MFLUCC 17–2091	MT214573	MT310618	–	MT394632
*Pleopunctum ellipsoideum*	MFLU 19–0685	MK804517	MK804512	–	MK828510
*Pleopunctum ellipsoideum*	KUNCC 10784	OQ146984	ON261161	–	OQ943188
*Pleopunctum ellipsoideum*	MFLUCC 24-0081	PP657324	PP657285	–	–
***Pleopunctum ellipsoideum***	**HKAS 144358**	**PX591017**	**PX591012**	–	**PX593725**
*Pleopunctum heveae*	MFLUCC 21-0146a	OL782070	OL780491	–	–
*Pleopunctum heveae*	MFLUCC 21-0146b	OL782071	OL780492	–	–
*Pleopunctum menglaense*	KUMCC 21–0025	ON009102	ON009118	–	ON009261
*Pleopunctum menglaense*	KUMCC 21–0026	ON009103	ON009119	–	ON009262
*Pleopunctum multicellularum*	KUNCC 10789	OQ146989	ON261166	–	OQ943190
*Pleopunctum multicellularum*	KUNCC 10781	OQ146981	ON261158	–	OQ943189
*Pleopunctum multicellularum*	KUNCC 10778	OQ146978	ON261155	–	–
*Pleopunctum thailandicum*	MFLUCC 21–0039	MZ198896	MZ198894	–	MZ172461
***Pleopunctum xizangense***	**HKAS 144353^T^**	**PX591018**	**PX591013**	–	**PX593726**
***Pleopunctum xizangense***	**HKAS 144354**	**PX591019**	**PX591014**	–	**PX593727**
*Pseudocapulatispora clematidis-subumbellatae*	MFLUCC 17-2063	MT214560	MT310607	MT226677	MT394739
*Pseudocapulatispora longiappendiculatum*	MFLUCC 17-1452^T^	MT214462	MT214368	MT214415	MT235783
*Pseudocapulatispora longiappendiculatum*	MFLUCC 17-1457	MT214463	MT214369	MT214416	MT235784
*Pseudolophiostoma lincangense*	KUNCC 21-0606^T^	PP779904	PP779895	PP779900	PP778369
*Pseudolophiostoma chiangraiense*	MFLUCC 17_2076^T^	MT214561	MT310608	MT226678	MT394740
*Pseudolophiostoma clematidis*	MFLUCC 17-2081	MT214562	MN393004	MT226679	MT394741
*Pseudolophiostoma cornisporum*	KH 322^T^	LC312544	LC312515	LC312486	LC312573
*Pseudolophiostoma mangiferae*	MFLUCC 17-2651^T^	MG931025	MG931031	MG931028	–
*Pseudolophiostoma mangiferae*	MFLUCC 17-2653	MG931026	MG931032	MG931029	–
*Pseudolophiostoma obtusisporum*	KT 3098	LC312548	LC312519	LC312490	LC312577
*Pseudolophiostoma obtusisporum*	KT 2838^T^	LC312547	LC312518	LC312489	LC312576
*Pseudolophiostoma tropicum*	KH 352	LC312550	LC312521	LC312492	LC312579
*Pseudolophiostoma tropicum*	KT 3134^T^	LC312551	LC312522	LC312493	LC312580
*Pseudolophiostoma vitigenum*	HH 26930^T^	AB619015	LC001735	AB618697	LC001761
*Pseudolophiostoma vitigenum*	HH 26931	AB619016	LC001736	AB618698	LC001762
***Pseudolophiostoma yunnanense***	**HKAS 144365^T^**	**PX591020**	**PX591015**	**PX591010**	**PX593728**
***Pseudolophiostoma yunnanense***	**HKAS 144366**	**PX591021**	**PX591016**	**PX591011**	**PX593729**
*Pseudopaucispora brunneospora*	KH 227^T^	LC312552	LC312523	LC312494	LC312581
*Pseudoplatystomum scabridisporum*	BCC 22835	GQ925844	–	–	GU479857
*Pseudoplatystomum scabridisporum*	BCC 22836	GQ925845	–	–	GU479856
*Quintaria lignatilis*	BCC 17444	GU479797	–	GU479764	GU479859
*Sigarispora coronillae*	MFLUCC 14-0941^T^	KT026112	KT026120	KT026116	–
*Sigarispora junci*	MFLUCC 14-0938^T^	MG829078	MG828966	MG829178	–
*Sigarispora ravennicum*	MFLUCC 14-0005^T^	KP698414	KP698413	KP698415	–
*Sigarispora scrophulariicola*	MFLUCC 17-0689^T^	MG829081	MG828969	–	–
*Teichospora rubriostiolata*	TR7	KU601590	KU601590	–	KU601609
*Teichospora trabicola*	C134	KU601591	KU601591	–	KU601601
*Thyridaria macrostomoides*	GKM1033	GU385190	–	–	GU327776
*Thyridaria macrostomoides*	GKM1159	GU385185	–	–	GU327778
*Thyridaria macrostomoides*	GKM224N	GU385191	–	–	GU327777
*Vaginatispora appendiculata*	MFLUCC 13-0835^T^	KY264745	–	KY264749	–
*Vaginatispora aquatica*	MFLUCC 11-0083^T^	KJ591576	KJ591577	KJ591575	–
*Vaginatispora scabrispora*	KT 2443^T^	LC312554	LC312525	LC312496	LC312583

Boldface: data generated in this study, ^T^ ex-type material, – means no data.

Maximum likelihood (ML) analysis was performed with RAxML-HPC2 on XSEDE v. 8.2.12 via the CIPRES Science Gateway v. 3.3 (https://www.phylo.org/), using the GTR+GAMMA+I model and 1,000 rapid bootstrap replicates. Evolutionary models for each barcode were estimated for Bayesian inference (BI) analysis using jModelTest v.2.1.10 (Guindon and Gascuel 2003; Darriba et al. 2012) based on the Akaike information criterion (AIC). Bayesian analysis was performed with MrBayes v. 3.2.7 (Ronquist et al. 2012). Bayesian posterior probability (BYPP) was evaluated using Markov Chain Monte Carlo (MCMC) sampling (Rannala and Yang 1996; Zhaxybayeva and Gogarten 2002). Six simultaneous Markov chains were run for 2,000,000 generations, and trees were sampled every 100 generations. The first 25% of trees, representing the burn-in phase of the analyses, were discarded, while the remaining 75% were used to calculate PP for the majority-rule consensus tree (Hua et al. 2006). The resulting trees were illustrated in FigTree v. 1.4.0 (http://tree.bio.ed.ac.uk/software/figtree) and edited in Adobe Illustrator 2020 (Adobe Inc., United States).

## Results

### Phylogenetic analyses

In this study, three taxa were collected from southwestern China. Two phylogenetic datasets were generated, and combined with morphological and phylogenetic assessment, it was determined that these taxa belonged to 2 genera and 2 families.

### Phaeoseptaceae

**Figure 1.**
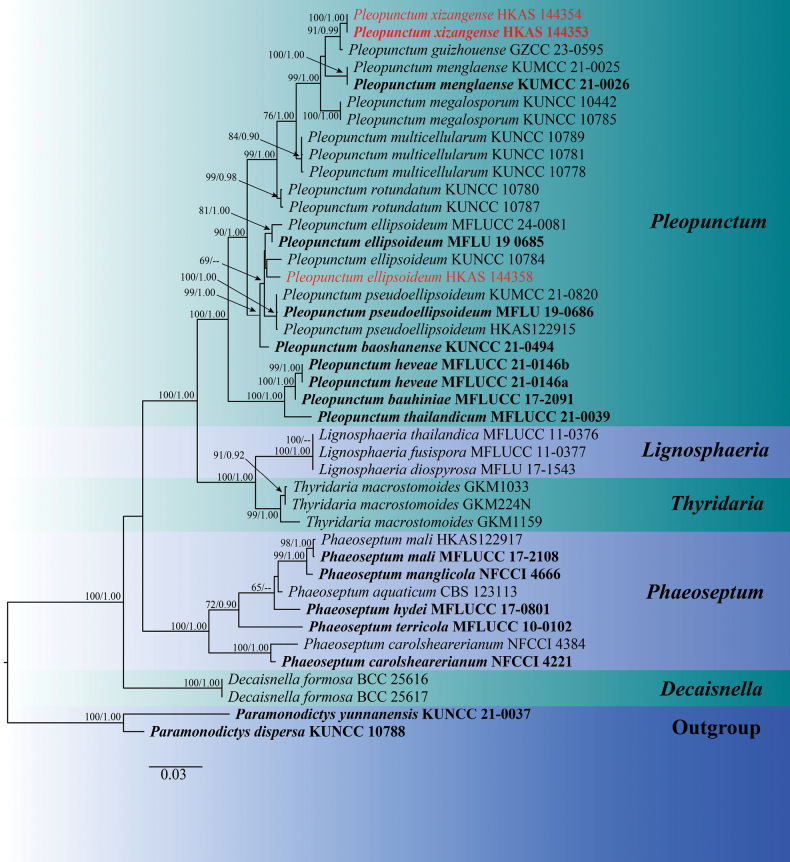
Maximum likelihood consensus tree of Phaeoseptaceae inferred from the combined ITS, LSU and *tef1* multiple sequence alignments. Bootstrap support values for maximum likelihood (ML, first value) equal to or greater than 65% and Bayesian posterior probabilities from MCMC analyses (BYPP, second value) equal to or greater than 0.90 are given above the nodes. The scale bar indicates 0.03 changes per site. The tree is rooted in *Paramonodictys dispersa* and *Paramonodictys yunnanensis*. The type strains are in bold, while the newly generated sequences are indicated in red.

Phylogenetic analyses of the combined ITS, LSU, and *tef1* dataset comprised 25 taxa, including *Paramonodictys dispersa* (KUNCC 10788) and *Paramonodictys yunnanensis* (KUNCC 21-0037) as outgroup taxa following Zhang et al. (2023). The dataset consisted of 2,257 total characters, including gaps (ITS: 1–829 bp, LSU: 830–1,357 bp, *tef1*: 1,358–2,257 bp). The matrix contained 728 distinct alignment patterns, with 15.46% of characters undetermined or missing. Estimated base frequencies were as follows: A = 0.227735, C = 0.275353, G = 0.281218, T = 0.215693; substitution rates: AC = 1.255123, AG = 2.600537, AT = 1.435453, CG = 1.208749, CT = 8.773403, GT = 1.000000; gamma distribution shape parameter α = 0.822366. The best-scoring RAxML tree with a final likelihood value of -10226.586190 is presented in Fig. 1.

### Pseudolophiostoma

**Figure 2.**
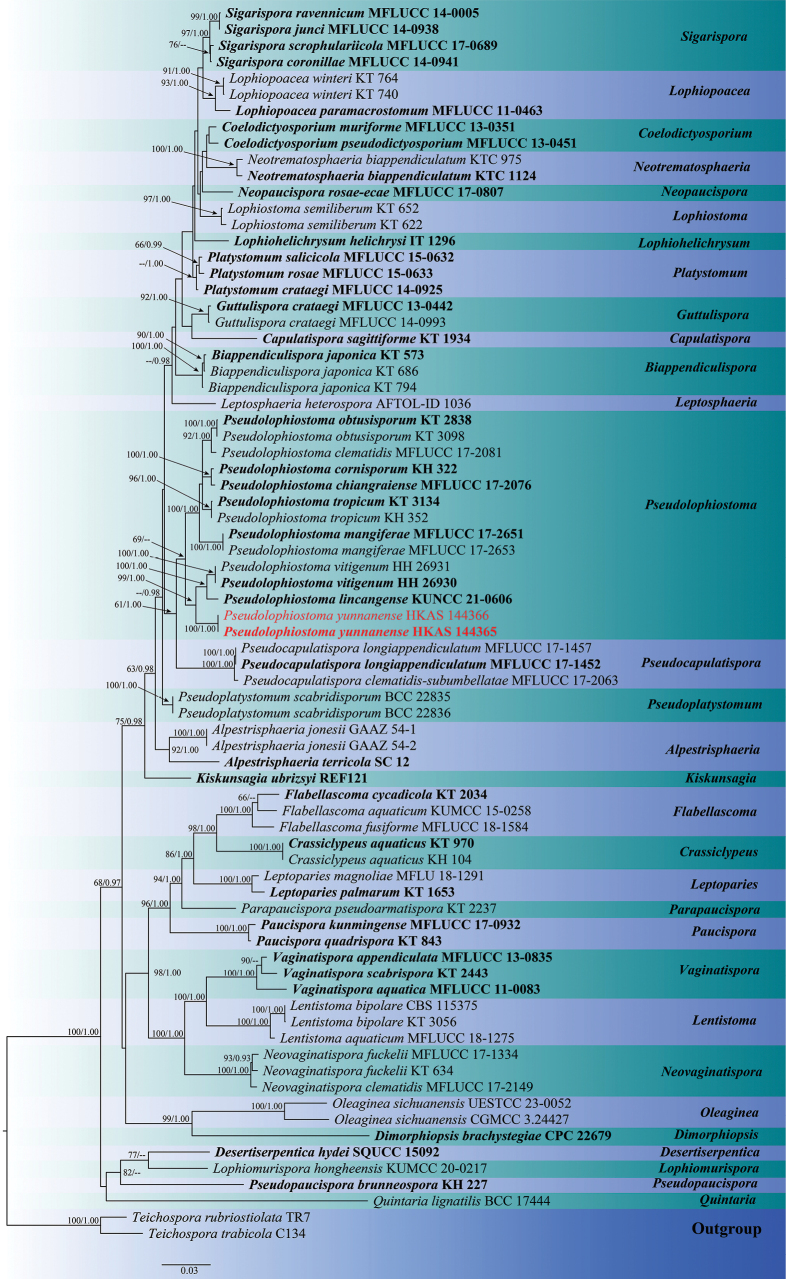
Maximum likelihood consensus tree of the family Lophiostomataceae inferred from the combined SSU, LSU, ITS and *tef1* multiple sequence alignments. Bootstrap support values for maximum likelihood (ML, first value) equal to or greater than 60% and Bayesian posterior probabilities from MCMC analyses (BYPP, second value) equal to or greater than 0.90 are given above the nodes. The scale bar indicates 0.03 changes per site. The tree is rooted in *Teichospora rubriostiolata* and *Teichospora trabicola*. The type strains are in bold, while the newly generated sequences are indicated in red.

Phylogenetic analyses of the combined SSU, LSU, ITS, and *tef1* dataset comprised 58 taxa, with *Teichospora rubriostiolata* (TR7) and *Teichospora trabicola* (C134) (Andreasen et al. 2021) as the outgroup taxa. The dataset consisted of 3,248 total characters, including gaps (SSU: 1–974 bp, ITS: 975–1,830 bp, LSU: 1,831–2,354 bp, *tef1*: 2,355–3,248 bp). The matrix contained 1,091 distinct alignment patterns, with 15.52% of characters undetermined or missing. Estimated base frequencies were as follows: A = 0.246385, C = 0.244585, G = 0.267520, T = 0.241511; substitution rates: AC = 1.477273, AG = 2.786449, AT = 1.381973, CG = 1.196495, CT = 7.496630, GT = 1.000000; gamma distribution shape parameter α = 0.521099. The best-scoring RAxML tree with a final likelihood value of -21384.045088 is presented in Fig. 2.

### Taxonomy

#### Pleopunctum xizangense

Taxon classificationFungiPleosporalesPhaeoseptaceae

Y.Y. Yang, K.D. Hyde & Q. Zhao sp. nov.

CCF21AF6-036E-51A5-8563-1F41C5496A1D

860714

Facesoffungi Number: FoF17374

Fig. 3

##### Etymology.

Refers to the type location, Xizang Autonomous Region, China.

**Figure 3.**
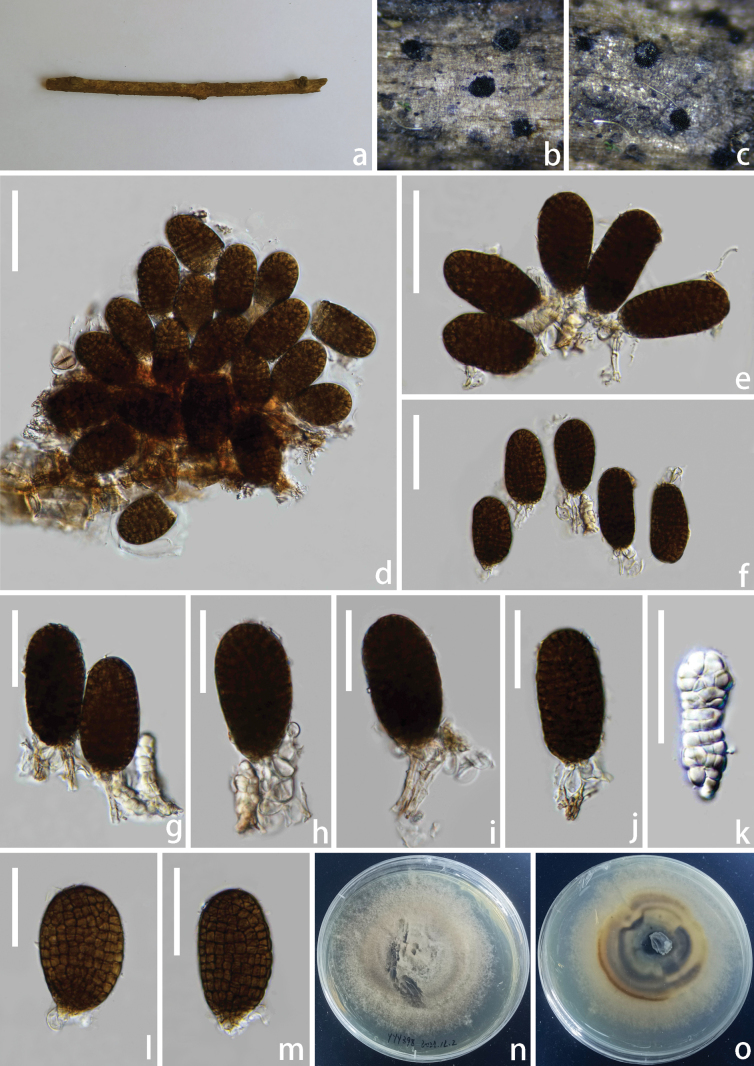
*Pleopunctum xizangense* (HKAS 144353, holotype). **a**. Natural substrate; **b, c**. Colonies on the substrate; **d–f**. Conidia mass; **g–j**. Conidiophores and conidia; **k**. Immature conidia; **l, m**. Conidia; **n, o**. Upper and lower views of cultures on PDA agar after incubation for 8 days. Scale bars: 60 μm (**d–f**); 40 μm (**g–m**).

##### Holotype.

HKAS 144353.

##### Description.

Saprobic on the dead branch of *Quercus* sp. **Sexual morph**: Undetermined. **Asexual morph**: Hyphomycetous. ***Colonies*** on natural substrate sporodochial, superficial, black, glistening, gregarious, and punctiform. ***Mycelium*** immersed in the substratum, composed of subhyaline to brown hyphae, septate, branched. ***Conidiophores*** macronematous, mononematous, cylindrical, smooth-walled, hyaline to pale brown, unbranched, septate, short, straight. ***Conidiogenous cells*** monoblastic, terminal, integrated, hyaline to pale brown. ***Conidia*** 55–70 × 29–36 µm (x = 63 × 32 µm, n = 30), solitary, acrogenous, oval to long elliptical, hyaline to pale brown when immature, brown to dark brown when mature, muriform, constricted at the septa, smooth-walled, broadly obtuse at apex, basal cell 4–15 × 2–8 µm (x = 9 × 5 µm, n = 25), 0–multiple-basal cell, hyaline, thick-walled, elliptical to globose.

##### Culture characteristics.

Conidia germinated on PDA media and produced germ tubes within 24 h. The colony reached 0.8 cm in diameter at 25 °C in the incubator for 8 days on PDA. Colonies grown on PDA are grey, subcircular, with dense, irregular margins, reverse grey at the centre, and pale brown at the margin.

##### Material examined.

China • Xizang Autonomous Region, Shigatse City, Nyalam County, on a dead branch of *Quercus* sp., 6 July 2022, Yanyan Yang, YYY398 (holotype: HKAS 144353), ex-type living culture KUNCC 25-191120; China • Xizang Autonomous Region, Shigatse City, Nyalam County, on a dead branch of *Quercus* sp., 6 July 2022, Yanyan Yang, YYY398-2 (HKAS 144354), living culture KUNCC 25-191121.

##### Notes.

In the LSU, ITS, and *tef1* combined phylogenetic analyses, our species formed a sister subclade to *P. guizhouense* (GZCC 23-0595) with 89 ML/1.00 PP statistical support. Sequence comparison for the ITS region between *P. xizangense* (HKAS 144353) and *P. guizhouense* (GZCC 23-0595) showed a 1.0% (489/494 bp) base pair difference and 0.6% (846/851 bp) base pair difference for the LSU region. Morphologically, *P. xizangense* (HKAS 144353) is similar to *P. guizhouense* (GZCC 23-0595) in having superficial and sporodochial colonies, septate conidiophores, monoblastic and terminal conidiogenous cells, and muriform, oval to ellipsoidal conidia. However, *P. xizangense* (HKAS 144353) differs from *P. guizhouense* in the size and shape of the basal cell. *Pleopunctum xizangense* (HKAS 144353) has 0–multiple-basal cells per conidia that are 4–15 × 2–8 µm. *Pleopunctum guizhouense* has an ellipsoidal to globose basal cell per conidia that are 16–26 × 11–17 µm. *Pleopunctum guizhouense* was collected on submerged decaying wood in freshwater from Guizhou Province, China, by Zhang et al. (2023), while our isolate was collected on a dead branch of *Quercus* sp. in terrestrial from Xizang Autonomous Region, China. Considering these morphological differences and their distinct phylogenetic positions, *P. xizangense* was introduced as a new species in *Pleopunctum*.

#### Pleopunctum ellipsoideum

Taxon classificationFungiPleosporalesPhaeoseptaceae

N.G. Liu, K.D. Hyde & J.K. Liu, in Liu, Hyde, Bhat, Jumpathong & Liu, Mycosphere 10(1): 768 (2019)

FBEFC1CC-BA26-5A17-987C-FC78FF52F2CC

Index Fungorum: IF556523

Facesoffungi Number: FoF06114

Fig. 4

##### Description.

Saprobic on the dead branch of *Quercus fabri*. **Sexual morph**: Undetermined. **Asexual morph**: Hyphomycetous. ***Colonies*** on natural substrate, superficial, black, glistening, gregarious, punctiform, sporodochial. ***Mycelium*** immersed in the substratum, composed of subhyaline to pale brown hyphae, septate, branched. ***Conidiophores*** macronematous, mononematous, cylindrical, hyaline to pale brown, simple, septate, thick-walled, short. ***Conidiogenous cells*** monoblastic, terminal, integrated, hyaline. ***Conidia*** 29–43 × 17–26 µm (x = 36 × 21 µm, n = 40), acrogenous, solitary, oval to ellipsoidal, broadly obtuse at apex, brown, muriform, constricted at the septa, sometimes truncate at base, smooth-walled, ellipsoidal to globose basal cell, 13–21 × 12–18 μm (x = 17 × 15 μm, n = 30).

**Figure 4.**
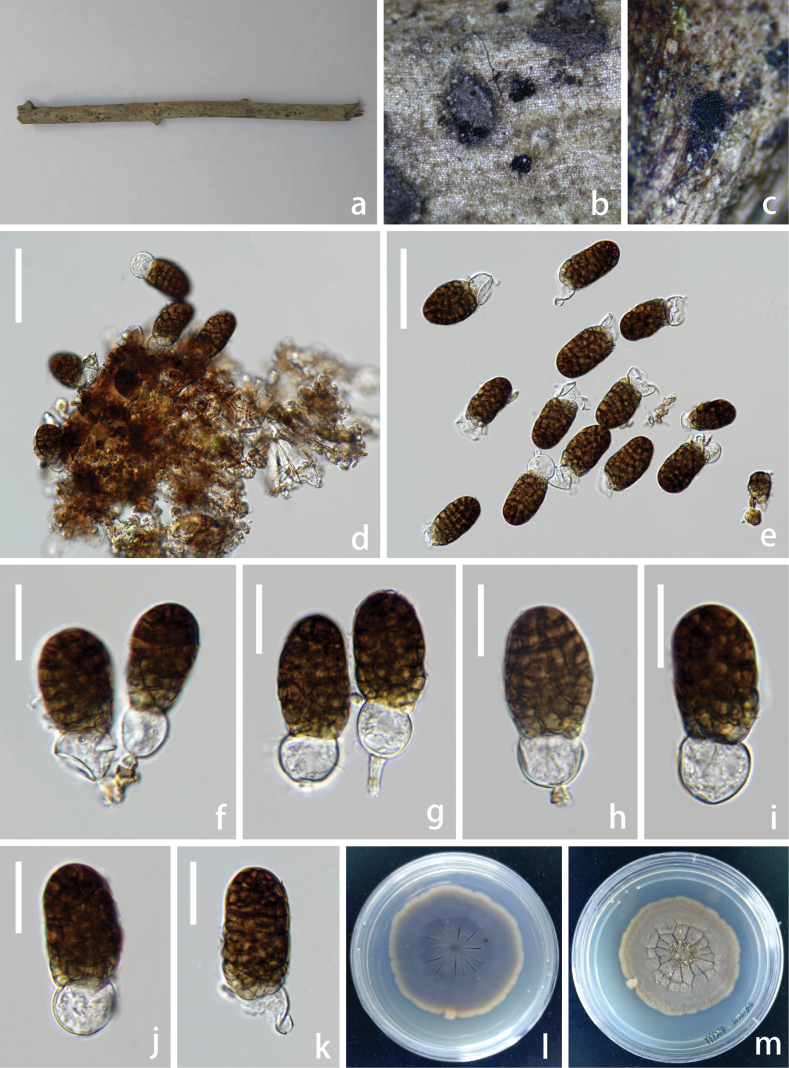
*Pleopunctum ellipsoideum* (HKAS 144358). **a**. Natural substrate; **b, c**. Colonies on the substrate; **d, e**. Conidia mass; **f–k**. Conidia with hyaline basal cells; **l, m**. Upper and lower views of cultures on PDA agar after incubation for 2 months. Scale bars: 50 μm (**d–e**); 20 μm (**f–k**).

##### Culture characteristics.

Conidia were germinated on PDA media and produced germ tubes within 24 h. The colony reached 5.6 cm in diameter at 25 °C in the incubator for 2 months on PDA. Colonies grown on PDA are greyish green, wrinkled, subcircular, mycelia dense, yellowish secreta in the centre, revers dark olive green, yellow at margin.

##### Material examined.

China • Yunnan Province, Kunming City, Panlong District, on a dead branch of *Quercus fabri*, 8 May 2022, Yanyan Yang, YYY234 (HKAS 144358), living culture KUNCC 25-19122.

##### Notes.

In the combined phylogenetic analyses (LSU, ITS, and *tef1*), our isolate (HKAS 144358) clustered with *Pleopunctum ellipsoideum* (MFLU 19-0685) with 92% ML/0.99 PP statistical support. The base pair comparison between our new isolate (HKAS 144358) and *P. ellipsoideum* (KUNCC 10784) revealed 1.4% (486/493 bp) for ITS, 0.5% (775/779 bp) for LSU, and 2.8% (733/754 bp) for *tef1*. Morphologically, our isolate resembles *P. ellipsoideum* (KUNCC 10784) in cylindrical, macronematous conidiophores, muriform, oval to ellipsoidal conidia with an elliptical to globose basal cell. However, *P. ellipsoideum* (HKAS 144358) differs from *P. ellipsoideum* (KUNCC 10784) in having larger basal cells (13–21 × 12–18 vs. 4–12 × 8–13 µm). Liu et al. (2019) reported *P. ellipsoideum* from unidentified decaying wood in Guizhou Province, China. Subsequently, Xu et al. (2023) reported *P. ellipsoideum* (KUNCC 10784) collected on submerged decaying wood in a freshwater from Yunnan Province, China. Our isolate was collected from a dead branch of *Quercus fabri* in a terrestrial habitat. This is the first of *P. ellipsoideum* on this host.

#### Pseudolophiostoma yunnanense

Taxon classificationFungiPleosporalesPhaeoseptaceae

Y.Y. Yang, K.D. Hyde & Q. Zhao sp. nov.

C222167C-334E-54DB-A6C4-EC5D871BAAB6

860715

Facesoffungi Number: FoF18345

Fig. 5

##### Etymology.

Refers to the type location, Yunnan Province, China.

**Figure 5.**
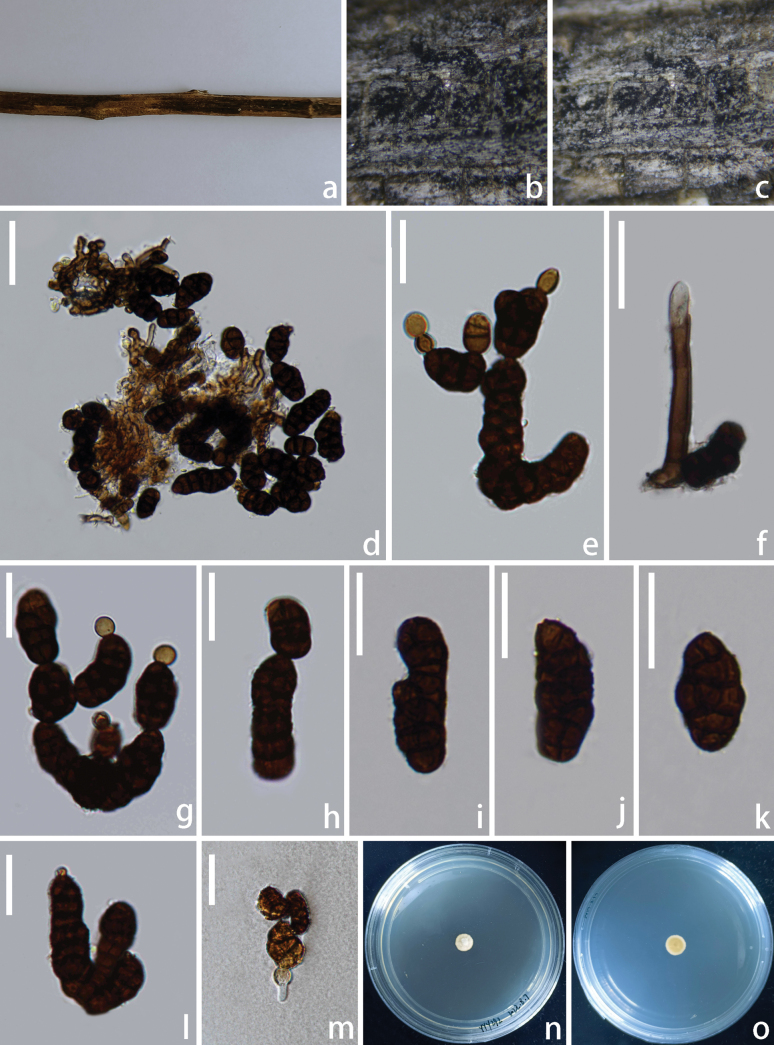
*Pseudolophiostoma yunnanense* (HKAS 144365, holotype). **a**. Natural substrate; **b, c**. Colonies on the substrate; **d**. Conidiogenous cells giving rise to conidia; **e, g, l**. Branched chains of conidia; **f**. Conidia with seta; **h–k**. Conidia; **m**. Germinated conidia; **n, o**. Upper and lower views of cultures on PDA agar after incubation for 23 days. Scale bars: 30 μm (**d, f, m**); 20 μm (**e, g–l**).

##### Holotype.

HKAS 144365.

##### Description.

Saprobic on the dead branch of *Alnus* sp. **Sexual morph**: Undetermined. **Asexual morph**: Hyphomycetous. ***Colonies*** on natural substrate superficial, black, powdery, gregarious, punctiform. Mycelium immersed in the substratum, composed of septate, branched, brown hyphae. ***Conidiophores*** macronematous, mononematous, simple, flexuous, branched, brown, thick-walled, septate, short. ***Setae*** scattered mixed with conidiophores, 67–95 µm long, 6–9 µm wide (x– = 78 × 7.5 µm, n = 8), dark brown, hyaline at the apex, straight, septate, solitary, with a long cylindrical stipe, smooth-walled. ***Conidiogenous cells*** monoblastic, terminal, integrated, brown. ***Conidia*** 15–43 × 10–21 µm (x = 27 × 14 µm, n = 40), acrogenous and pleurogenous, catenate, thick-walled, muriform, constricted at septa, pale brown when young, dark brown when mature, cylindrical to cylindric-clavate.

##### Culture characteristics.

Conidia were germinated on PDA media and produced germ tubes within 24 h. The colony reached 1 cm in diameter at 25 °C in the incubator for 23 days on PDA. Colonies grown on PDA are white, round, dense mycelia, soft mycelium, reverse pale yellow, and white at the margin.

##### Material examined.

China • Yunnan Province, Nujiang Lisu Autonomous Prefecture, Gongshan Derung and Nu Autonomous County, on a dead branch of *Alnus* sp., 16 July 2022, Yanyan Yang, YYY292 (holotype: HKAS 144365), ex-type living culture KUNCC25-20217; China • Yunnan Province, Nujiang Lisu Autonomous Prefecture, Gongshan Derung and Nu Autonomous County, on a dead branch of *Alnus* sp., 16 July 2022, Yanyan Yang, YYY292-2 (HKAS 144366), living culture KUNCC25-20218.

##### Notes.

In the multi-gene phylogenetic analysis, *Ps. yunnanense* formed a distinct clade within Lophiostomataceae and formed a sister clade to *Ps. vitigenum* and *Ps. lincangense* with 99 ML/1.00 PP statistical support. The base pair comparisons showed that *Ps. yunnanense* differed from *Ps. vitigenum* by 0.89% (780/787 bp), 5.75% (459/487 bp), 0.20% (957/959 bp), and 4.33% (795/831 bp) in the LSU, ITS, SSU, and *tef1*, respectively, and from *Ps. lincangense* by 1.1% (778/787 bp), 6.3% (456/487 bp), 0% (960/960 bp) and 3.8% (799/831 bp) in the LSU, ITS, SSU, and *tef1*, respectively. Morphologically, *Ps. yunnanense* is hyphomycetous, characterized by dark brown and scattered setae, muriform and catenate conidia. The asexual morph of *Pseudolophiostoma* has never been reported. We introduce *Ps. yunnanense* as a new species in *Pseudolophiostoma*, based on analyses of LSU, ITS, SSU, and *tef1* gene sequences. In addition, we provide a description and photographic plates in this study.

## Discussion

In this study, we describe two new taxa and a new host record from dead branches in terrestrial habitats of Yunnan Province and Xizang Autonomous Region, China, namely *Pleopunctum xizangense*, *P. ellipsoideum*, and *Pseudolophiostoma yunnanense*. These genera are widely distributed and reported from terrestrial and freshwater habitats worldwide, whereas *Pleopunctum* has been reported from terrestrial and freshwater habitats in China and Thailand (Hyde et al. 2020; Jayawardena et al. 2022; Zhang et al. 2023). Many species in these genera exhibit a broad host range, and here we report, for the first time, *P. xizangense*, *P. ellipsoideum*, and *Ps. yunnanense* on Fagales hosts.

Phaeoseptaceae previously accommodated three genera, *Phaeoseptum*, *Lignosphaeria*, and *Neolophiostoma* (Hyde et al. 2018). Liu et al. (2019) introduced *Pleopunctum* in Phaeoseptaceae, and *Neolophiostoma* was transferred to Halotthiaceae by Liu et al. (2019) based on phylogenetic analysis. According to Hongsanan et al. (2020) and Wijayawardene et al. (2022), Phaeoseptaceae has only two genera, i.e., *Phaeoseptum* and *Pleopunctum*. In phylogenetic analyses, the genera of *Decaisnella*, *Lignosphaeria*, and *Thyridaria* also clustered within Phaeoseptaceae (Hyde et al. 2018, 2020; Wanasinghe et al. 2020, 2022; Xu et al. 2023). Therefore, the type species of these genera need to be re-collected and re-examined to ensure the correct placement (Abdel-Wahab and Jones 2003; Mugambi and Huhndorf 2009; Hyde et al. 2018; Wanasinghe et al. 2022).

Currently, Index Fungorum (http://www.indexfungorum.org, accessed 15 September 2025) lists eight names under the genus *Pseudolophiostoma*, viz. *Ps. chiangraiense*, *Ps. clematidis*, *Ps. cornisporum*, *Ps. lincangense*, *Ps. mangiferae*, *Ps. obtusisporum*, *Ps. tropicum*, and *Ps. vitigenum*. However, *Pseudolophiostoma clematidis*, *Ps. cornisporum*, *Ps. obtusisporum*, and *Ps. tropicum* were transferred to *Lophiostoma* by Andreasen et al. (2021), whereas *Ps. chiangraiense* was subsequently transferred to the same genus by Li et al. (2023). Nevertheless, in our phylogenetic tree, all these taxa cluster within the *Pseudolophiostoma* clade. *Pseudolophiostoma* is typified by *Pseudolophiostoma vitigenum*, which was previously described as *Lophiotrema*. *Lophiostoma* is a speciose genus with numerous species and is polyphyletic (Zhang et al. 2009). Thambugala et al. (2015) divided *Lophiostoma* into 16 genera based on the multi-locus phylogenies (LSU, SSU, and *tef1*) (Hashimoto et al. 2018). Andreasen et al. (2021) resurrected the generic concept of *Lophiostoma*, and 14 genera were synonymized under *Lophiostoma* based on morphology and molecular phylogeny (ITS2, LSU, *tef1*, and RPB2). However, the species classification of Lophiostomataceae is not fully resolved by phylogenetic reconstruction. In our phylogenetic tree, some main stems have low support, possibly because many Lophiostomataceae species lack RPB2 sequences (Fig. 3). Therefore, adding RPB2 and *tef1* sequences could strengthen support for deeper nodes and improve the topology. Currently, *Lophiostoma* has 496 entries in Index Fungorum (http://www.indexfungorum.org, accessed 15 September 2025), but many species lack molecular evidence.

## Supplementary Material

XML Treatment for Pleopunctum xizangense

XML Treatment for Pleopunctum ellipsoideum

XML Treatment for Pseudolophiostoma yunnanense

## Additional information

### Use of AI

No use of AI was reported.

### Author ORCIDs

Yan-Yan Yang https://orcid.org/0009-0007-9521-558X

Tai-Shun Li https://orcid.org/0000-0001-8697-1630

Vinodhini Thiyagaraja https://orcid.org/0000-0002-8091-4579

Chitrabhanu S. Bhunjun https://orcid.org/0000-0001-8098-3390

Shu-Cheng He https://orcid.org/0009-0008-7364-4727

Pattana Kakumyan https://orcid.org/0000-0001-8281-1082

Qi Zhao https://orcid.org/0000-0001-8169-0573

Kevin D. Hyde https://orcid.org/0000-0002-2191-0762

### Data availability

All of the data that support the findings of this study are available in the main text.
